# Correction: Food security and nutritional vulnerability in Comoros: The impact of Russia-Ukraine conflict

**DOI:** 10.1371/journal.pone.0323506

**Published:** 2025-05-02

**Authors:** Estefanía Custodio, Maria Priscila Ramos, Sofía Jimenez, Francis Mulangu, Nicolas Depetris-Chauvin

S2 and S3 Files are uploaded incorrectly. Please see the correct S2 and S3 Files here.

## Supporting information

S2 File Read me file for Comoros nutrients composition table. (TXT)

S3 File Comoros nutrients composition table. (XLSX)

## References

[1] Custodio E, Ramos MP, Jimenez S, Mulangu F, Depetris-Chauvin N. Food security and nutritional vulnerability in Comoros: The impact of Russia-Ukraine conflict. PLoS One. 2024;19(11):e0313388. doi: 10.1371/journal.pone.0313388 39531483 PMC11556715

