# Supplementary material for: Food security and nutritional vulnerability in Comoros: The impact of Russia-Ukraine conflict
Source: PLoS One. 2024 Nov 12;19(11):e0313388. doi: 10.1371/journal.pone.0313388 (PMC11556715; doi:10.1371/journal.pone.0313388)
Supplement: S1 File — (DOCX) [file pone.0313388.s001.docx]

**Appendix**

**Table A.1. Estimated demand price elasticities by food groups in Comoros**

| Diversity food groups | | Estimated Price-Demand elasticities |
| --- | --- | --- |
| 1 | Cereals | -2.706 |
| 2 | White Roots & Tubers | -0.595 |
| 3 | Vit. A Rich Vegetables & Tubers | -0.51 |
| 4 | Dark Green Leafy Vegetables | -0.341 |
| 5 | Other Vegetables | -0.345 |
| 6 | Vit. A Rich Fruits | -0.238 |
| 7 | Other Fruits | -0.555 |
| 8 | Organ Meat | -1.592 |
| 9 | Flesh Meats | -1.592 |
| 10 | Eggs | -0.17 |
| 11 | Fish & Seafood | -0.251 |
| 12 | Legumes, Nuts & Seeds | -1.925 |
| 13 | Milk & Milk Products | -0.964 |
| 14 | Oils & Fats | -0.267 |
| 15 | Sweets | -0.74 |
| 16 | Spices, Condiments, Beverages | -0.598 |

Source: own estimations based on the Enquête Harmonisée sur les Conditions de Vie des Ménages 2018/2019 – EHCVM 2020 and INSEED’s price indexes (2022-2023).

**Table A.2. Recommendation of daily calories and nutrients intakes per capita.**

| **Food Consumption** | | | **Recommendations** |
| --- | --- | --- | --- |
| Food sufficiency | | Energy intake | 2,250 kcal |
|  |  |  |  |
| Food Adequacy | Macronutrients | Fat kcal (share) | 0.15 - 0.3 |
|  |  |  |  |
|  |  | Protein (share) | 0.10 - 0.15 |
|  |  |  |  |
|  |  | Carbohydrates (share) | 0.55 - 0.75 |
|  |  |  |  |
|  | Micronutrients | Calcium intake | 1000 mg |
|  |  | Iron intake | 6.0 mg |
|  |  | Zinc intake | 9.4 mcg |
|  |  | Folate intake | 320 mcg |
|  |  | VitARAE intake | 625- mcg |
|  |  | Betacarotene intake |  |
|  |  | VitB1 intake | 2 mcg |
|  |  | VitB2 intake | 1.1 mg |
|  |  | VitC intake | 75 mg |

Source: own elaboration base on KNBS (2018), FAO and WHO (2003) recommendations.

**Table A.3. Prevalence of food sufficiency and adequacy of the diet across Comorian households (% of all households) – comparison before and after (one year) the beginning of the Ukraine-Russia conflict.**

|  | National | | Rural | | Urban | |
| --- | --- | --- | --- | --- | --- | --- |
|  | Before | After | Before | After | Before | After |
|  | The beginning of the U-R war | | | | | |
| Food sufficiency |  |  |  |  |  |  |
| DEC per capita per day >= 2,250 kcal | 42% | 39% | 39% | 36% | 47% | 44% |
| Food adequacy (macronutrients) |  |  |  |  |  |  |
| A balanced diet | 11% | 16% | 11% | 16% | 11% | 16% |
| An unbalanced diet | 33% | 32% | 33% | 31% | 34% | 33% |
| Protein share < 10% | 13% | 12% | 15% | 13% | 10% | 9% |
| Fat share < 15% | 2% | 3% | 2% | 3% | 2% | 3% |
| Carbohydrate share < 55% | 78% | 69% | 79% | 68% | 77% | 71% |
| Protein share > 15% | 36% | 41% | 33% | 39% | 42% | 46% |
| Fat share > 30% | 70% | 59% | 71% | 59% | 68% | 60% |
| Carbohydrate share > 75% | 1% | 1% | 1% | 1% | 0% | 1% |

Source: own elaboration.

Note: The threshold for food sufficiency is a daily caloric requirement of 2,250 kilocalories. A balanced diet means that recommended goals for macronutrients are all simultaneously met. An unbalanced diet implies not meeting at least one of the WHO macronutrient intake proportions (in deficit or in excess).
