# Supplementary material for: Food security and nutritional vulnerability in Comoros: The impact of Russia-Ukraine conflict
Source: PLoS One. 2024 Nov 12;19(11):e0313388. doi: 10.1371/journal.pone.0313388 (PMC11556715; doi:10.1371/journal.pone.0313388)
Supplement: Attachment — Submitted filename: PLS_review.docx [file pone.0313388.s005.docx]

Review on “Food security and nutritional vulnerability in small island developing states (SIDS): Analyzing diet adequacy and the impact of Ukraine-Russia conflict in food security and nutrition indicators in Comoros”

Summary

This paper examines food and nutritional security in Comoros which is one of the least studies small island developing states. Moreover, the authors examine the effects of Ukraine-Russia War on the food and nutritional security, by using simulations. They found that Comoros experiences limited access to sufficient and nutritious diets, and a decline in food access and purchasing power for all households, particularly the poorer and rural. Their contribution is to fill a crucial gap in the literature by examining the impact on nutrients, providing essential information for targeted policy recommendations. They suggest that policies promoting the consumption of fresh, nutrient-rich foods with low fat content are crucial to mitigate the malnutrition triple burden.

This paper uses solid descriptive analysis to answer their research questions.

I ask the authors to share the Appendix for a further review.

Major comments

1. Regarding the literature about Ukraine-Russian War and food security, you may want to include the following papers as past studies.

   Trade disruption

Lin, F., Li, X., Jia, N., Feng, F., Huang, H., Huang, J., ... & Song, X. P. (2023). The impact of Russia-Ukraine conflict on global food security. Global Food Security, 36, 100661.

Alexander, P., Arneth, A., Henry, R., Maire, J., Rabin, S., & Rounsevell, M. D. (2023).

Worsen cost of agricultural input
High energy and fertilizer prices are more damaging than food export curtailment from Ukraine and Russia for food prices, health and the environment. Nature Food, 4(1), 84-95.

2. In Equation (1), why do you add negative sign in the equation?
3. Line 216, where does ε show up in equations? How did you translate the changes in food prices into changes in food quantities, busing the estimates of price-demand elasticities?
4. How did you derive “the mean amount of fat consumed in Comoros is 91 grams per day per capita, which closely aligns with the mean fat intake in Fiji and Guam Island, countries with some of the highest obesity rates globally”? Table 1 shows the mean fat intake from 797 to 869 kcal per day per capita. I suggest that you include the value of kilogram in a table to easily understand the statement.
5. Line 360, according to Figure 3B, households below 40 % of per capita expenditure increased their food consumption. How do you interpret the result?
6. The authors argue the need to promote local fresh fruits and vegetables production and consumption. However, the results show that Ukraine-Russian war has positively affected on food adequacy except for Folate. I know the Comoros needs more improvement in food and nutrition intake but it does not sound the implication derived from the analysis. I suggest that the authors rewrite their implication. This is a surprising result for me.
7. Line 435, for example, “the conflict's effects resulted in a notable reduction in iron intake for all households” is strong conclusion because 40% of households experience increase in Iron intake. The authors need to rewrite the conclusion.

Minor comments

- Line 91, delete r between average and SDG2.
- Line 99, delete e between by and the.
- Line 113, delete average.
- Line 115, delete but.
