# Supplementary material for: Food security and nutritional vulnerability in Comoros: The impact of Russia-Ukraine conflict
Source: PLoS One. 2024 Nov 12;19(11):e0313388. doi: 10.1371/journal.pone.0313388 (PMC11556715; doi:10.1371/journal.pone.0313388)
Supplement: Attachment — Submitted filename: Response to Reviewers.docx [file pone.0313388.s006.docx]

**PLOS ONE**

10^th^ of September 2024

Academic Editor of PLOS ONE

Subject: Original Research Article REVISION

Dear Prof. Dr. Abu Hayat Md. Saiful Islam,

On August 14^th^, 2024, we received a revised decision letter for our submitted paper "Food security and nutritional vulnerability in small island developing states: Analyzing diet adequacy and the impact of Ukraine-Russia conflict in food security and nutrition indicators in Comoros" (Ref. PONE-D-24-27344). We would like to thank you for this opportunity, and for the reception of yours and the reviewers’ valuable comments, which have been very useful for improving the original manuscript.

In the following pages, we provide replies to all the comments raised by both you (the academic editor) and the reviewers. These changes are marked in track changes in the manuscript. We also provide a clean version of the manuscript to facilitate reading.

We hope that this new version of the article is improved to your satisfaction, and that it reaches the high-quality standards for being considered for publication in PLOS ONE.

Yours faithfully,

the authors.

**Academic Editor**

1. Please ensure that your manuscript meets PLOS ONE's style requirements, including those for file naming. The PLOS ONE style templates can be found at <https://journals.plos.org/plosone/s/file?id=wjVg/PLOSOne_formatting_sample_main_body.pdf>
We have edited the text to follow the style requirements described in the document.

and <https://journals.plos.org/plosone/s/file?id=ba62/PLOSOne_formatting_sample_title_authors_affiliations.pdf>
We have edited the authors affiliations according to the guidelines.

2. Please note that PLOS ONE has specific guidelines on code sharing for submissions in which author-generated code underpins the findings in the manuscript. In these cases, we expect all author-generated code to be made available without restrictions upon publication of the work. Please review our guidelines at https://journals.plos.org/plosone/s/materials-and-software-sharing#loc-sharing-code and ensure that your code is shared in a way that follows best practice and facilitates reproducibility and reuse.

We include in this submission the State code and data properly labeled and explained.

3. In the online submission form, you indicated that the data of the Comoros National Household survey is available upon request from the Nataional Statistics Institute of Comoros (INSEED), at INSEED's website https://www.inseed-comores.org/category/enquetes/ehcvm-2020/. The Comoros Nutrients Composition Table elaborated by the authors will be held in a public repository (RepiSalud)

All PLOS journals now require all data underlying the findings described in their manuscript to be freely available to other researchers, either 1. In a public repository, 2. Within the manuscript itself, or 3. Uploaded as supplementary information.

This policy applies to all data except where public deposition would breach compliance with the protocol approved by your research ethics board. If your data cannot be made publicly available for ethical or legal reasons (e.g., public availability would compromise patient privacy), please explain your reasons on resubmission and your exemption request will be escalated for approval.

We cannot make the data publicly available for legal reasons. The data belong to the National Statistics Institute of Comoros (INSEED), and only them can give access to the data. We, as researchers, made the request to obtain the data for analysis but not to make them publicly available. However, the data is available from the INSEED upon request. All the other data in the analysis is provided in the submission.

4. We note that you have included the phrase “data not shown” in your manuscript. Unfortunately, this does not meet our data sharing requirements. PLOS does not permit references to inaccessible data. We require that authors provide all relevant data within the paper, Supporting Information files, or in an acceptable, public repository. Please add a citation to support this phrase or upload the data that corresponds with these findings to a stable repository (such as Figshare or Dryad) and provide and URLs, DOIs, or accession numbers that may be used to access these data. Or, if the data are not a core part of the research being presented in your study, we ask that you remove the phrase that refers to these data.

These data were complementary, but not a core part of the research. Thus, we have removed the phrase referring to these data.

5. Please amend either the title on the online submission form (via Edit Submission) or the title in the manuscript so that they are identical.

As suggested by one of the reviewers, we have changed the title in the manuscript to “Food security and nutritional vulnerability in Comoros: the impact of Ukraine-Russia-Ukraine conflict”. Accordingly, we have updated the title in the online submission form, thus, they are identical now.

**Reviewer: 1**

This paper examines food and nutritional security in Comoros which is one of the least studies small island developing states. Moreover, the authors examine the effects of Ukraine-Russia War on the food and nutritional security, by using simulations. They found that Comoros experiences limited access to sufficient and nutritious diets, and a decline in food access and purchasing power for all households, particularly the poorer and rural. Their contribution is to fill a crucial gap in the literature by examining the impact on nutrients, providing essential information for targeted policy recommendations. They suggest that policies promoting the consumption of fresh, nutrient-rich foods with low fat content are crucial to mitigate the malnutrition triple burden.
This paper uses solid descriptive analysis to answer their research questions.
I ask the authors to share the Appendix for a further review.

We would first like to thank the reviewer for his/her positive assessment of our work and for his/her comments, which we believe helped to improve the manuscript. Below, we explain the changes made in the manuscript to address them. Besides, we attach the Appendix with all the supplementary material.

1. Regarding the literature about Ukraine-Russian War and food security, you may want to include the following papers as past studies.

Trade disruption

Lin, F., Li, X., Jia, N., Feng, F., Huang, H., Huang, J., ... & Song, X. P. (2023). The impact of Russia-Ukraine conflict on global food security. Global Food Security, 36, 100661.

Worsen cost of agricultural input

Alexander, P., Arneth, A., Henry, R., Maire, J., Rabin, S., & Rounsevell, M. D. (2023). High energy and fertilizer prices are more damaging than food export curtailment from Ukraine and Russia for food prices, health and the environment. Nature Food, 4(1), 84-95.

Both papers are already included in the paper. Particularly, they are included in the following paragraphs of the introduction:

“Moreover, countries in Sub-Saharan Africa, such as Tanzania or Kenya, have especially experienced high inflation rates due to this conflict, which has impacted strongly on the welfare of its population (Lin et al., 2023; Glauben et al., 2022).”

“…Firstly, it directly reduces the production of agricultural commodities worldwide. Secondly, it leads to an increase in transportation costs for food and fertilizers (Alexander et al., 2023; Hatab, 2022; Lin et al., 2023).”

Besides, we have corrected the first reference and included all the authors in the bibliography.

2. In Equation (1), why do you add negative sign in the equation?

This is customary in the trade and welfare literature: an increase in prices reduces welfare. We have explained that in the text now.

3. Line 216, where does ε show up in equations? How did you translate the changes in food prices into changes in food quantities, busing the estimates of price-demand elasticities?

Yes, we use the price-demand elasticities. We have explained that in the text now.

4. How did you derive “the mean amount of fat consumed in Comoros is 91 grams per day per capita, which closely aligns with the mean fat intake in Fiji and Guam Island, countries with some of the highest obesity rates globally”? Table 1 shows the mean fat intake from 797 to 869 kcal per day per capita. I suggest that you include the value of kilogram in a table to easily understand the statement.

The mean amount of fat consumed in Comoros is derived from applying the Atwater factor (9 for fats), to the mean calories consumed (822 kcal). That is, we divide 822 by 9=91.3 grams. We have added a footnote to Table 1 for clarification.

5. Line 360, according to Figure 3B, households below 40 % of per capita expenditure increased their food consumption. How do you interpret the result?

Thank you for pointing this out. We did not comment on this result, but our interpretation is that the	Russia-Ukraine conflict	impacted primarily the prices of imported and expensive food products like organ meats, nuts, and imported fish and seafood	negatively affecting the diet of the wealthier	population. On the other hand, prices of red meat, sweets and oils and fats decreased (See Figure 2 of the manuscript) being more affordable for poorer households. And these groups are the ones that contribute more to the energy intake, as shown in Table 2. Therefore, the daily energy consumption (DEC) was affected differently for poorer and wealthier households. We have added this interpretation to the text.

6. The authors argue the need to promote local fresh fruits and vegetables production and consumption. However, the results show that Ukraine-Russian war has positively affected on food adequacy except for Folate. I know the Comoros needs more improvement in food and nutrition intake but it does not sound the implication derived from the analysis. I suggest that the authors rewrite their implication. This is a surprising result for me.

Indeed, according to our simulations the Ukraine-Russian war had positively affected some measures of food adequacy. This result is driven by changes in relative prices. The war has affected more the price of imported food products which in normal times are cheaper but with less nutritional value. The substitution effect would direct households to consume more local products with better nutritional value at the cost of affecting food sufficiency. Moreover, our approach is partial equilibrium. In reality, in a general equilibrium setting, this increased in demand for local food products may result in further increased in the price of these products if their supply is relatively inelastic. Therefore, it is important to promote the conditions that will support a more elastic response, including a sustained local demand.

We have clarified that now in the text.

7. Line 435, for example, “the conflict's effects resulted in a notable reduction in iron intake for all households” is strong conclusion because 40% of households experience increase in Iron intake. The authors need to rewrite the conclusion.

Indeed, we have amended the text accordingly. As stated in the text we interpret the reduction in iron intake in wealthier households due to the reduction of expensive products like organ meats and nuts (not accessible for poorer households before the conflict) and increase in iron intake of poorer households with the decrease in price and increase consumption of red meat.

Minor comments

Line 91, delete r between average and SDG2.

Line 99, delete e between by and the.

Line 113, delete average.

Line 115, delete but.

Thanks, we have corrected all these typos.

**Reviewer: 2**
Comoros amidst the Russia-Ukraine conflict. As a reviewer, my comments are as follows:

General comments: I think the insights produced in this manuscript will be helpful for policy formulation in Comoros. It is a timely and demand-driven research. Without a few punctuational mistakes, I found the manuscript well-written.

We would first like to thank the reviewer for his/her positive assessment of our work and for his/her comments, which we believe helped to improve the manuscript. Below, we explain the changes made in the manuscript to address them.

1. Title: I recommend the title should be “Food Security and Nutritional Vulnerability in Comoros: The Impact of Russia-Ukraine Conflict”.

Following the reviewer's suggestion, we have changed the title to “Food Security and Nutritional Vulnerability in Comoros: The Impact of Russia-Ukraine Conflict”.

2. Affiliation: If it is possible, write the affiliation in International Language for the general readers.

Thanks for the comment. All the affiliations are written in International Language now.

3. Abstract:

Ø The Sustainable Development Goal #2 aims to eradicate hunger. Avoid # sing.

We have revised the manuscript and avoid # sign in the text.

4. Keywords: I recommend the keywords and the order is as follows-

Ø Food security, Nutrition, Diet adequacy, Russia-Ukraine Conflict, Comoros

We have modified the key words as suggested by the reviewer.

5. Introduction:

Ø The introduction is a bit large. To avoid it, authors may keep some parts of it in the footnote. For instance, lines 50 to 52.

Ø I found both Russia-Ukraine and Ukraine-Russia in the manuscript. To maintain homogeneity, I suggest Russia-Ukraine.

Ø Lines 63 to 64 are not clear. Please, rewrite.

Ø In line 67, change the word provision to production.

Ø From lines 77 to 94, readers will lose readability. Keep some in the footnote.

Ø The average r SDG2 score. Is it ok? Check.

Ø Line 99, exacerbated byethe COVID-19 pandemic. Please, check.

Ø Lines 103 to 104. Check.

Ø Line 115. Check.

Ø Line 120, change provision to production.

Thanks for the deep review of the introduction. We have shortened the section and rewritten some parts. We have also checked all the typos highlighted by the reviewer.

6. Methodology:

Ø Sufficiently explained.

7. Results:

Ø This part is also well explained.

8. Discussion and conclusion:

Ø In this part, I found some redundancy. For instance, from lines 418 to 446. I am not convinced by the present discussion. Please, rewrite by avoiding redundancy.

Ø I recommend a separate conclusion section.

Ø The conclusion is also poor. Please rewrite.

We agree with the reviewer, the conclusion as it was presented was somehow repetitive and there was not enough emphasis on the main points. We have rewritten the whole section.
